# Cytological and Gene Expression Analysis Reveal Salt Resistance in Common Wild Rice (*Oryza rufipogon* Griff.): A Case for Root Tissue Resistance

**DOI:** 10.3390/plants15152247

**Published:** 2026-07-23

**Authors:** Yutong Zheng, Wenjia Huang, Xinghui Xie, Shihui Chen, Zhiyan Liu, Zhongxi Wu, Xinyi Zeng, Xiangdong Liu, Jinwen Wu

**Affiliations:** 1 Rice Germplasm Resource Bank of Guangdong Province (SCAU), South China Agricultural University, Guangzhou 510642, China; zhyutong@stu.scau.edu.cn (Y.Z.); jin_aoba@stu.scau.edu.cn (W.H.); xinhuixie@stu.scau.edu.cn (X.X.); shchen@stu.scau.edu.cn (S.C.); lzyl@stu.scau.edu.cn (Z.L.); a3466529023@stu.scau.edu.cn (Z.W.); zuobiaozhou@stu.scau.edu.cn (X.Z.); xdliu@scau.edu.cn (X.L.); 2 Guangdong Provincial Key Laboratory of Plant Molecular Breeding, South China Agricultural University, Guangzhou 510642, China; 3 Base Bank of Lingnan Rice Germplasm Resources, College of Agriculture, South China Agricultural University, Guangzhou 510642, China

**Keywords:** common wild rice, salt stress, root tissue, genetic variation

## Abstract

Common wild rice (*Oryza rufipogon* Griff.) is an important germplasm resource harboring valuable genes. Our previous analysis identified a core collection of wild rice primarily originating from the common wild rice of Guangdong Province. However, there was no information on its salt-stress tolerance. In this study, we assessed 13 accessions of common wild rice collected in Gaozhou, Guangdong, China. Salt-stress treatments applied at the tillering and seedling stages revealed that CRW3 exhibited stronger salt tolerance than CRW13 under NaCl treatment. Cytological observations indicated that root tip elongation in CRW3 was less inhibited by NaCl treatment, accompanied by a lower percentage of abnormalities in cortical cells and layers, as well as reduced shrinkage of epidermal cells. Resequencing analysis identified 2390 genes with genetic variations between CRW3 and CRW13. Furthermore, RT-qPCR was conducted to examine the expression levels of salt-stress-related genes in these two accessions. Overall, our study demonstrates the strong salt tolerance of CRW3, providing a theoretical foundation for the selection of salt-tolerant germplasm in rice.

## 1. Introduction

Rice (*Oryza sativa* L.) is a staple food for more than half of the global population. In recent years, rice production has been increasingly constrained by population growth, shrinking arable land, environmental degradation, and soil salinization [1,2,3]. Among these, soil salinization is a major challenge to food security and has severely hindered sustainable agricultural development. Hence, there is an urgent need to develop salt-tolerant varieties and identify novel salt-resistance genes to enhance yield, improve grain quality, and strengthen stress tolerance [4]. Salt stress impairs root development in rice through a combination of osmotic and ionic disturbances, which, in turn, compromise water and nutrient uptake and transport, ultimately inhibiting plant growth and development [5,6]. Compared with rice cultivars, wild rice species harbor a rich array of stress-resistance genes and offer a promising resource for addressing these challenges [7]. Moreover, they possess numerous beneficial alleles absent in domesticated germplasm, making them an invaluable genetic reservoir for rice breeding [8,9].

Common wild rice (*Oryza rufipogon* Griff.) is considered the ancestor of Asian cultivated rice. In China, it is one of three wild rice species and is predominantly distributed in Guangdong, Hainan, Guangxi, and Yunnan provinces. This wild rice exhibits considerable genetic divergence from cultivated rice and displays stronger resistance to diseases, pests, and abiotic stresses [9,10]. These traits are essential for improving stress tolerance in rice cultivars. Through interspecific hybridization, several beneficial traits, such as tolerance to aluminum toxicity, cold resistance, blast resistance, and drought adaptation, have been successfully incorporated into rice cultivars through interspecific hybridization. To date, numerous resistance genes conferring resistance to biotic and abiotic stresses have been identified in common wild rice [11,12,13]. Genetic populations derived from wild rice, including inbred and introgression lines, are frequently used to identify candidate resistance genes [14,15,16]. Two salt-tolerance quantitative trait loci (QTLs), *qST1.2* and *qST6*, have been identified from introgression lines of Dongxiang wild rice [17].

High-throughput sequencing techniques, such as whole-genome resequencing and RNA-seq, are essential for identifying genetic variants and differentially expressed genes (DEGs). Whole-genome resequencing facilitates the discovery of functional genes and promotes crop improvement [18]. It can identify sequence polymorphisms, including single-nucleotide polymorphisms (SNPs) and insertions/deletions (InDels) [19,20,21]. Combined with phenotypic evaluation, sequencing technologies have been used to uncover genetic variation loci associated with agronomic traits [22,23,24,25]. Wild crop species are valuable genetic resources, and their genomic data can enhance the genetic diversity of cultivated varieties [26]. Research into wild rice germplasm through genome sequencing, along with the identification and utilization of its elite genes, opens new avenues for rice breeding. A “wild rice–cultivated rice pan-genome” has been constructed, revealing 28,907 core genes and 13,728 wild-rice-specific genes [27]. Whole-genome sequencing of a Dongxiang wild rice population showed that half of the SNPs and InDels are absent in cultivated rice and other wild rice populations [28]. Moreover, several genes and QTLs exhibit differential expression during population differentiation. These findings will broaden the genetic resource pool for rice and contribute to improving the quality of rice varieties [29,30,31].

Common wild rice from Guangdong Province has been shown to harbor diverse stress-resistance genes and represents a valuable germplasm resource for rice breeding [32,33,34]. In this study, we selected 13 accessions of wild rice, primarily from Gaozhou City in Guangdong Province. We conducted a preliminary NaCl stress experiment using tiller stems to evaluate the salt tolerance of these materials. Furthermore, we employed cytological and resequencing analyses to confirm the strong salt tolerance of CRW3, examine patterns of genomic variation, and identify potential genetic variants associated with salt tolerance. This integrated approach, combining phenotypic, cytological, and genomic analyses, helps elucidate the molecular basis of salt tolerance in wild rice species.

## 2. Results

### 2.1. Effects of NaCl Stress on Tiller Stem Elongation in Common Wild Rice

To investigate the salt tolerance of common wild rice from Gaozhou, Guangdong Province, tiller stems of 13 accessions were collected and assessed for their responses to a high salt concentration (150 mmol/L NaCl). In this study, relative root elongation (RRE) of tiller stems was used as an indicator to quantify genetic variation in salt tolerance (Figure 1). Among the tested materials, CRW3 displayed the least inhibition and sustained normal root growth under saline conditions, whereas CRW13 was the most severely affected. At 150 mmol/L NaCl, the RRE values for CRW3 were 0.26 ± 0.03 after 7 days of treatment, whereas CRW13 exhibited a markedly lower RRE of 0.03 ± 0.01 under the same conditions. The difference in RRE between CRW3 and CRW13 was significant. These findings indicate that CRW3 exhibited a higher RRE than the other materials.

### 2.2. Effects of NaCl Stress on the Root Morphology in Common Wild Rice

Among the 13 wild rice accessions, CRW3 and CRW13 exhibited the highest and lowest levels of salt tolerance ability, respectively. In this study, we compared the root morphologies of these two materials under NaCl stress (Figure 2). Salt stress significantly affected root growth in both wild rice varieties. Under salt stress, root elongation of CRW13 almost completely stopped after 3–5 days (Figure 2H,I), and root growth showed no significant increase even after 7 days (Figure 2J). In contrast, although root growth in CRW3 was hindered under salt stress, it continued to increase steadily (Figure 2E). Ultimately, root elongation in CRW3 was significantly greater and more stable than that in the salt-sensitive CRW13. These results indicated that the roots of CRW3 could maintain stable growth and exhibit strong adaptability under salt stress.

### 2.3. Effects of Root Morphology on Reactive Oxygen Species Under NaCl Stress

We compared the root staining patterns of CRW13 and CRW3 using Nitro Blue Tetrazolium (NBT) staining (Figure 3). Excessive ROS accumulation was closely linked to impaired plant growth. Under normal conditions, the root tips of both CRW13 and CRW3 were clearly visible and showed only light staining, indicating very low levels of superoxide anions (Figure 3A,C). The mature zones and lateral roots appeared healthy, indicating a good growth status (Figure 3E,G).

Under salt stress, both CRW13 and CRW3 exhibited increased accumulation of superoxide anion (O_2_^−^) at the root tips compared with the control group (Figure 3B,D). In the salt-tolerant CRW3, the root tips stained deep purple, indicating substantial superoxide anion accumulation, while the mature zone and lateral roots remained light purple, although lateral root growth was markedly inhibited (Figure 3F). These observations suggest that salt stress suppresses lateral root development and induces superoxide anion accumulation. Overall, the roots of CRW13 exhibited darker staining, higher ROS levels, and consequently greater oxidative stress than those of CRW3.

To better illustrate the differing reactions of the two lines under salt stress, physiological indicators, including soluble sugar content and CAT and POD activities in root tips, were measured (Table S1). The results showed that both materials accumulated soluble sugars and increased POD activity, while their CAT activities declined relative to the control under oxidative stress. CRW13 accumulated more soluble sugars and exhibited a greater decrease in CAT activity, resulting in weaker antioxidant capacity. Compared with CRW13, CRW3 accumulated less soluble sugar and maintained higher CAT activity, thereby sustaining a superior antioxidant balance under salt stress.

### 2.4. Comparison of Root Elongation and Increased Root Number of Gaozhou Wild Rice Under Different NaCl Stress Concentrations

To characterize differential root responses between the salt-tolerant line (CRW3) and the salt-sensitive line (CRW13) under various NaCl stress levels, we used a series of NaCl concentration gradients to examine the salt-stress effects on root growth. Root length, relative root elongation rate, and root count increment were quantified for the two materials in this study.

Root length, relative root elongation, and root count increment indicate root system growth and development. In this study, we measured the root lengths of CRW3 and CRW13 across various NaCl concentrations (Figure 4A,B). There were no significant differences in root lengths between CRW13 and CRW3 at 0 and 50 mmol/L. For both materials, root lengths increased over time and eventually exceeded those of the control group. However, at NaCl concentrations of 100 and 150 mmol/L, root elongation was suppressed in both variants.

In this study, the root elongation of CRW13 was more severely inhibited under 100 mmol/L NaCl (Figure 4B). Root length showed only a slight increase early on, measuring 1.95 ± 0.41 cm on the 3rd day, 2.52 ± 0.18 cm on the 5th day, and 2.64 ± 0.17 cm on the 7th day, indicating minimal elongation. When the NaCl concentration was increased to 150 mmol/L, root elongation was completely suppressed. Although the root lengths were 1.39 ± 0.42 cm on the 3rd day, 1.41 ± 0.46 cm on the 5th day, and 1.42 ± 0.45 cm on the 7th day, no significant growth was detected over this period. Compared with salt-sensitive CRW13, the salt-tolerant CRW3 exhibited a stable growth trend under 100 mmol/L NaCl (Figure 4A). The root lengths were 2.89 ± 0.40 cm, 4.29 ± 0.82 cm, and 5.79 ± 1.27 cm on the 3rd, 5th, and 7th days, respectively. Under 150 mmol/L NaCl, the root lengths on the 3rd, 5th, and 7th days were 2.28 ± 0.82 cm, 3.57 ± 1.11 cm, and 4.76 ± 1.04 cm, respectively. These results indicate that CRW13 is more sensitive to NaCl stress, and its root elongation is more markedly inhibited.

Additionally, the dynamic changes in relative root elongation between the two materials at different NaCl concentrations are summarized (Figure 4C,D). In this study, both materials, CRW3 and CRW13, showed a decreasing trend in relative root elongation as NaCl concentration and treatment duration increased. However, at a low NaCl concentration (50 mmol/L), the root elongation of CRW3 exhibited a relative growth rate higher than that of the control during the initial 3 days, whereas that of CRW13 showed a similar trend but with less pronounced growth. The relative root elongation values of CRW3 and CRW13 were 1.26 ± 0.22 and 1.01 ± 0.11, respectively. At higher NaCl concentrations (100 mmol/L and 150 mmol/L), root elongation in both accessions was inhibited, with CRW13 showing a greater decrease and severe inhibition of root growth, whereas CRW3 still maintained some growth capacity. After 7 days of exposure to 150 mmol/L NaCl, the relative root elongation values of CRW3 and CRW13 were 0.36 ± 0.04 and 0.04 ± 0.01, respectively. These findings demonstrate that CRW3 exhibited markedly greater salt tolerance than CRW13.

The number of newly formed roots under NaCl stress can serve as a measure of salt tolerance in the salt-tolerant (CRW3) and salt-sensitive (CRW13) materials. In this study, we investigated the increase in root number across different NaCl concentrations for both materials (Figure 4E,F). As the NaCl concentration increased, the root length of both materials decreased. For CRW13, the number of newly formed roots differed significantly among the 50, 100, and 150 mmol/L NaCl treatments. Specifically, the values were 3.06 ± 0.34, 3.00 ± 0.50, and 1.73 ± 0.51, respectively (Figure 4F). In comparison, CRW3 exhibited minimal variation in the number of newly formed roots across the same NaCl concentrations: 2.78 ± 0.22, 2.62 ± 0.23, and 2.22 ± 0.59, respectively (Figure 4E). These findings demonstrate that CRW13 is more sensitive to salt stress, whereas CRW3 exhibited greater stability under the salinity conditions.

### 2.5. Comparison of the Seeding Stage of Gaozhou Wild Rice Under Different NaCl Stress Concentrations

To verify the reliability of the preliminary screening, we also performed the NaCl treatment experiment during the seedling stage. The experiment during the rice seedling stage effectively demonstrates the variation in salt tolerance among different accessions. In the control groups, there were no significant differences in plant phenotype and growth between CRW3 and CRW13 (Figure 5A–C). With NaCl treatment, both materials began showing salt-injury symptoms at 100 mmol/L (Figure 5D–F). The leaf yellowing and wilting were significantly more severe in CRW13 than in CRW3 at 3 days of treatment (Figure 5E). On the 7th day, CRW13 exhibited pronounced withering, while CRW3 still maintained some green leaves despite experiencing partial wilting. Visually, CRW3 maintained superior growth under salt stress (Figure 5F).

CRW3 and CRW13 showed no significant differences in seedling growth traits such as shoot length, root length, and root number under control conditions and maintained normal growth (Figure 5G–I). Under the 100 mmol/L NaCl stress, differences between the two accessions gradually appeared. Shoot length in both CRW3 and CRW13 remained largely unchanged compared with pre-treatment levels, although CRW13 experienced a slight decrease, indicating restricted aboveground growth and wilting (Figure 5G). CRW3 maintained the ability to elongate its roots under stress, with root length measuring 8.58 ± 0.53 cm before NaCl stress treatment (0 d) and increasing to 9.22 ± 0.82 cm after 7 days. In comparison, root length in CRW13 was suppressed; its roots did not grow, resulting in no significant change (Figure 5H). Regarding root number, the increase in CRW3 was less affected by salt stress at 100 mmol/L NaCl (Figure 5I). Compared with CRW3, the increase in the root number of CRW13 was completely suppressed. Taken together, these indicators suggest that CRW3 can maintain a more stable root system architecture, thereby adapting to high-salt environments. The seedling growth of CRW3 under high-salt stress is significantly better than that of CRW13, indicating stronger salt tolerance.

### 2.6. Comparison of the Root Tips of CRW3 and CRW13 by WE-CLSM Analysis

To evaluate differences in root tip structures between salt-tolerant (CRW3) and salt-sensitive (CRW13) materials, we conducted WE-CLSM analysis in this study. The root tip structures of two materials, CRW3 and CRW13, were observed under normal conditions (CK) and following treatment with 150 mmol/L NaCl on the 7th day (Figure 6).

Under no-treatment growth conditions, the root tips of both CRW3 and CRW13 remained intact, with well-organized cells in the meristematic and elongation zones, clear outlines, and distinct cell layers (Figure 6A,C). The axial length of cortical cells in the mature root zone of CRW3 and CRW13 was 46.69 ± 5.79 μm and 48.89 ± 9.34 μm, respectively (Table S2). Under 150 mmol/L NaCl stress concentration, the root tip of CRW13 showed blurred cell outlines, indistinct boundaries between the meristematic and elongation zones, and some cells appeared shrunken or deformed, indicating disruption of root tip cell structure (Figure 6(D,D1)). In the mature zone, cell structures were severely impaired, with disorganized cell arrangement, destroyed intercellular spaces, and partial cell shrinkage; the axial length of cortical cells was 17.34 ± 2.42 μm compared with the control group (Figure 6(H,H1), Table S2).

Under the treatment of 150 mmol/L NaCl stress concentration, the root tip of CRW3 remained intact, although some cells appeared shrunken (Figure 6(B,B1)). The distinction between the meristematic and elongation zones in CRW3 also remained relatively clear. In the mature zone of CRW3, partial cells presented with shrinkage and deformation, and epidermal cells were shrunken and impaired, while the overall cellular architecture remained stable; compared with the control group, the axial length of cortical cells was 39.24 ± 4.63 μm (Figure 6(F,F1), Table S2). These results indicated that salt stress damages root tip architecture and suppresses cortical cell axial elongation. Under 150 mmol/L NaCl treatment, CRW13 exhibited severe root tip injury with a substantial reduction in cortical axial length, while CRW3 maintained a relatively intact root structure with only a slight decrease in cortical cell length.

### 2.7. Comparison of the Root Tissue of CRW3 and CRW13 Through Semi-Section Analysis

To further verify differences in root tissue zones between the salt-tolerant (CRW3) and salt-sensitive (CRW13) materials, we conducted a semi-section analysis. Root tip samples from these two materials were collected under normal conditions (CK) and after treatment with 150 mmol/L NaCl on the 7th day (Figure 7 and Figure 8).

Under no-treatment conditions, both CRW3 and CRW13 exhibited comparable intact root tip structures across all tissue regions. The root cap consisted of small, densely packed cells of uniform size (Figure 7A,C). The meristematic zone contained compact, regularly shaped cells with distinct tissue layers (Figure 7E,G). In the elongation zone, the epidermis, cortex, and stele were clearly demarcated; cortical cells were arranged in a regular radial pattern, and early differentiated vascular bundles were visible (Figure 8A,C). The number of cortical parenchyma cell layers or stele diameter within this zone in CRW3 and CRW13 showed no significant differences (Table S2). In the mature zone, the epidermis, cortex, and stele were well separated; cortical cells were uniformly shaped yet loosely organized, and the stelar vascular bundles contained neatly arranged xylem vessels and parenchyma cells with normal morphology (Figure 8E,G). In contrast, quantitative analysis of the mature zone revealed significant differences between the two lines in both cortical parenchyma cell layer numbers and stele diameters (Table S2).

When NaCl was increased to 150 mmol/L, the root cap cells of CRW3 appeared similar to those in the control group, showing no noticeable differences (Figure 7(B,B1)). In the meristematic zone, the overall cellular structure remained intact, and no obvious alterations were observed in cell morphology. (Figure 7(F,F1)). In the elongation zone, the number of cortical parenchyma cell layers decreased, and the stele diameter was reduced. Some endodermis and pericycle cells shrank; the overall tissue structure remained intact (Figure 8(B,B1)). In the mature zone, no obvious change was found in the number of cortical parenchyma cell layers, the stele diameter was reduced, and the boundary between the endodermis and stele became distinct (Figure 8(F,F1)).

The results showed that under normal conditions, CRW3 and CRW13 exhibited no significant differences in the overall root anatomical structure. However, CRW3 possessed a significantly higher number of cortical parenchyma cell layers in the mature zone compared with CRW13. After 150 mmol/L NaCl treatment, the root tissue structure of CRW13 was severely damaged, accompanied by an increase in the number of cortical parenchyma cell layers. In contrast, CRW3 maintained relatively intact root tissue structure and only displayed a reduced stele diameter in the elongation and mature zones.

### 2.8. Analysis of Genome Variation and Differential Gene Expression Between CRW13 and CRW3

To explore the genetic differences related to salt tolerance between CRW3 and CRW13 under NaCl treatment, we examined genome-wide single-nucleotide polymorphisms (SNPs) and insertions/deletions (InDels) (Figure 9). The results indicated that chromosome 1 had the largest number of single-nucleotide polymorphisms (SNPs), whereas chromosome 9 had the fewest (Figure 9B). A similar trend was observed for insertions/deletions (InDels), with the highest count on chromosome 1 and the lowest on chromosome 9 (Figure 9A). Here, we focused on two types of genetic variation in CRW3: non-synonymous SNPs and InDels within the coding sequence (CDS), as these are more likely to influence gene expression levels. Variations in this region can directly alter amino acid sequences, representing critical genetic variants that determine protein structure and biological function. We further performed differential analysis on CDS-derived SNPs and InDels between the two accessions. Compared with CRW13, a total of 2390 genes carried prominent sequence variations in CRW3 (Figure 9C).

To further characterize these differentially expressed genes (DEGs), GO enrichment analysis was performed. These divergent genes were mainly enriched in the three primary GO categories: biological process, molecular function, and cellular component (Figure 9E). In the biological process category, they were mainly enriched for pathways involved in response to stimulus and basal metabolism, including the nucleic acid metabolic process, among others (Table S3). In the molecular function category, they were significantly enriched for endopeptidase inhibitor activity and multiple binding capacities, as well as for DNA-binding transcription factor activity and sulfotransferase activity (Table S4). In the cellular component category, the DEGs were primarily localized to structures such as the Prp19 complex, the U2-type catalytic step 2 spliceosome, the extracellular matrix, the nucleus, and the thylakoid lumen (Table S5). Meanwhile, KEGG pathway enrichment analysis was also conducted in this study. The divergent genes revealed significant enrichment in multiple pathways associated with stress responses, secondary metabolism, and fundamental biological processes (Figure 9D). Additionally, plant–pathogen interaction, a pathway linked to plant stress resistance, was also significantly enriched. Several secondary metabolic pathways were significantly enriched, including flavonoid biosynthesis and sesquiterpenoid biosynthesis. These results indicate that CRW3 differs from CRW13 in the above-mentioned pathways and functions.

We integrated 2390 DEGs with known salt-tolerant and salt-sensitive genes and identified nine salt-stress-related genes that exhibited sequence variations in their coding sequences (Figure 10A, Table S6). Among these genes, four salt-stress-related candidate genes, named *LOC_Os08g33670*, *LOC_Os01g37870*, *LOC_Os01g03549*, and *LOC_Os07g40730*, were verified by RT-qPCR analysis (Figure 10B–E). In addition, we also selected eight cloned salt-stress-related genes for expression analysis: *LOC_Os03g13140*, *LOC_Os05g48700*, *LOC_Os06g27760*, *LOC_Os07g05940*, *LOC_Os02g52780*, *LOC_Os07g48130*, *LOC_Os09g13570*, and *LOC_Os12g42280* (Figure S1). After 7 days of 150 mmol/L NaCl treatment, the expression levels of these genes in the roots of CRW3 were upregulated compared with the normal control (CK). These results indicate that the expression patterns of these genes differ between CRW3 and CRW13 under salt stress, further demonstrating the difference in salt tolerance between the two materials.

## 3. Discussion

### 3.1. The Utilization of Wild Common Rice for the Detection of Salt-Stress Material

Rice is one of the most important staple crops worldwide [35,36,37]. However, soil salinization and ecological degradation increasingly threaten agricultural productivity and global food security [1]. Salt stress impairs root development in rice by reducing growth rate, decreasing root number, and diminishing root surface area and volume, ultimately resulting in shorter total root length. Therefore, enhancing salt tolerance in rice is critical to sustaining production. Common wild rice, the progenitor of cultivar rice, harbors extensive genetic diversity and exhibits high environmental adaptability, making it a valuable genetic resource for rice breeding [8]. Previous studies have shown that c from wild rice can markedly improve the salt tolerance of cultivated varieties [17]. Nevertheless, research into the root-specific salt-tolerance mechanisms in common wild rice remains limited. Elucidating these root-level responses is essential to advance the utilization of wild rice germplasm in salt-tolerance breeding. In this study, two common wild rice accessions, CRW3 and CRW13, which exhibit contrasting salt tolerance, were selected to investigate salt-induced changes in root morphology and physiology.

Plant development exhibits plasticity, enabling adaptation to environmental changes. The root system and associated tissues can adjust to salt stress [36]. In rice, root morphological parameters typically exhibit a biphasic response across varying NaCl concentrations, with promotion at low concentrations and inhibition at high concentrations [37,38]. Furthermore, salt-tolerant rice varieties tend to show greater root elongation under saline conditions [39]. In the present study, the root length and relative root elongation of tillers in CRW3 and CRW13 were similar to those in a previous study, even as NaCl concentration increased. Compared with CRW13, CRW3 exhibited less inhibition of root length and relative root elongation under NaCl stress, indicating its superior salt tolerance. In autotetraploid rice, salt-tolerant genotypes have been reported to maintain greater stability across a range of NaCl concentrations [37]. Our results further revealed that the increase in root number from CRW3 tillers remained relatively stable, whereas CRW13 was considerably more sensitive to salt stress.

Reactive oxygen species (ROS) play an important role in plant growth, development, and stress responses. Salt stress increased superoxide anion levels in plants. With increasing NaCl concentration, superoxide anions accumulated in rice roots. Nitro Blue Tetrazolium (NBT) reacts with superoxide anions (O_2_) to form a blue formazan. Areas with superoxide produce a blue-to-deep-blue stain, indicating the accumulation of reactive oxygen species (ROS). NBT staining can reflect ROS levels, with salt-sensitive lines exhibiting higher ROS accumulation than salt-tolerant lines [8,40,41]. In this study, the NBT staining method was used to compare the distribution of superoxide anions in the roots of CRW3 and CRW13. The root tips and lateral roots of both lines were lightly stained, indicating low ROS levels under normal conditions. Compared with the dark color of the salt-sensitive material in CRW13, CRW3 showed light purple staining in the mature zone and lateral roots.

Salt stress impacted rice seedling leaves, leading to yellowing, burning, and tip curling, and also affected root development [14,42]. Salt stress also significantly affects biomass-related traits in rice, notably by markedly inhibiting shoot length. The shoot length of common wild rice decreases, but its root dry weight is less affected under salt stress [14]. In this study, seedlings of two common wild rice lines were subjected to salt stress to validate the experiment. Compared with CRW13, CRW3 showed a slight decrease in shoot length but an increase in root length and root number, with the root system maintaining a certain capacity for growth. These results indicate that CRW3 showed stronger salt tolerance than CRW13.

### 3.2. CRW3 Is a Salt-Resistant Common Wild Rice

Salinization is the accumulation of high concentrations of sodium chloride and magnesium salts in soil and water [43]. Plant root tissues are directly exposed to salt stress and respond accordingly. Maintaining the structure of the root tip is vital for the plant’s ability to repair stress-induced damage, and regeneration helps repair mechanical damage caused by environmental conditions and abiotic stress [44]. Salt-tolerant varieties experience smaller decreases in total root length and surface area under salt stress, along with enhanced root adaptability [45]. The root tip has four zones: root cap, meristematic, elongation, and maturation. The root cap defends the root. The meristematic zone involves active cell division, with cells moving to the transition zone to elongate slowly. Then cells enter the elongation zone, where they elongate more rapidly, before reaching the maturation zone, where growth ceases [46,47]. Salt stress reduces cell volume, resulting in shrunken cells and disorganized rice root tips [48]. In this study, we compared the root anatomy of two wild rice accessions—salt-tolerant CRW3 and salt-sensitive CRW13—under control conditions and 150 mmol/L NaCl treatment for 7 days. CRW3 maintained a well-preserved root cap, meristematic, and elongation zones, with only a smaller stele diameter in the elongation and maturation zones under salt-stress treatment. Conversely, CRW13 exhibited significant structural disorganization, including blurred meristematic boundaries, shrunken epidermal cells, stele disruption, and vascular damage. These differences suggest that CRW3 can maintain the root tip structure despite salinity.

Whole-genome resequencing is a crucial method for identifying genetic differences in rice, providing new understanding of sequence polymorphisms through SNPs and InDels [49,50]. In this study, CRW3 and CRW13 were chosen from the available wild rice resources. To reveal the relationship between genome variation and differential salt response, we performed resequencing and identified 2390 genes with sequence variation between the two accessions. GO and KEGG enrichment analyses of these variant genes revealed significant enrichment in enzyme activities, transcriptional regulation, stress response, and secondary metabolism pathways between CRW3 and CRW13. This genomic evidence implies that CRW3 has evolved a complex regulatory network to cope with salt stress.

Until now, numerous salt-stress-associated genes have been reported [5,51,52]. This study examined the expression levels of 12 genes associated with salt tolerance in CRW3 and CRW13 [53,54,55,56,57,58,59,60,61,62,63,64]. *ONAC106* (*LOC_Os08g33670*) positively regulates salt tolerance in rice [53]. Under salt stress, this gene is upregulated in CRW3 yet downregulated in CRW13, with its expression pattern consistent with the salt-tolerance phenotypes of the two rice accessions. *OsLPR2* (*LOC_Os01g03549*) negatively regulates salt tolerance in rice [54]. Under salt stress, the transcript level of this gene remains largely stable in CRW3 but is significantly elevated in CRW13, suggesting that CRW3 can repress the aberrant activation of this negative regulator to prevent impairment of salt-tolerance pathways. *OsMSRB1.1* (*LOC_Os06g27760*) is upregulated under abiotic stresses such as salt and low temperature, enhancing oxidative stress tolerance [57]. Under the 150 mmol/L NaCl treatment, its expression level was upregulated in CRW3 but downregulated in CRW13 relative to the control. These results suggest that CRW3 probably activates salt-stress response pathways more rapidly and efficiently through the upregulation of salt-tolerance-related genes, thereby maintaining normal root growth. In addition, we also examined the expression level of salt-sensitive genes. The potassium transporter *OsHAK9* (*LOC_Os07g48130*) is a key positive regulator during seed germination under salt stress in rice [58]. *OsNCED5* (*LOC_Os12g42280*) is upregulated under salt stress [59]. Under the 150 mmol/L NaCl treatment, both genes were upregulated in CRW3 but significantly downregulated in CRW13 compared with the control. These findings indicate that CRW3 responds more sensitively to salt stress by rapidly inducing a regulatory network of relevant genes to cope with salt stress. Collectively, the differential expression of these genes between CRW3 and CRW13 under salt stress reveals the basis for CRW3’s stronger salt tolerance and provides a foundation for further dissection of its salt-tolerance mechanism.

## 4. Materials and Methods

### 4.1. Plant Materials

This study used 13 common wild rice (*Oryza rufipogon* Griff.) samples from Gaozhou City, Guangdong Province. Materials are stored at the Base Bank of Guangdong Rice Germplasm Resources at the South China Agricultural University.

### 4.2. Salt-Stress Treatment of Tillers in Common Wild Rice

To investigate genetic variation among common wild rice materials under salt stress, tiller stems at the vegetative growth stage were collected. All tiller stems were soaked in water for 1–2 days to maintain their growth vigor and promote root regeneration. Afterward, the stems were separated and submerged in rice nutrient solution for 24 h. Each treatment group was set up with three biological replicates. The five longest new roots per stem were measured under individual conditions.

### 4.3. Measurement of Root Tissue in Common Wild Rice

Three root-tissue-related traits, including root length, increased root number, and relative root elongation (RRE), were selected to investigate phenotypic differences at different NaCl concentrations. The root length and the number of tiller stems on newly emerged roots were measured at 1, 3, 5, and 7 days during the treatment. Relative root elongation (RRE) is the ratio of root growth under salt stress (0 and 150 mmol·L^−1^) to the control group’s growth at the same time points, calculated as RRE = (Root elongation under the NaCl stress treatment)/(Root elongation of the control). Each group was set up with three biological replicates, and each stem was measured by 3–5 roots, with the control group set up with biological replicates synchronously [65].

### 4.4. NBT Staining Analysis

Root samples from the salt-stress treatments were collected on the 7th day and immersed in ethanol. The samples were then immersed in the NBT staining solution (0.5 mg/mL, pH 7.8; Coolaber, Beijing, China, Cat# SL1806) and incubated at room temperature (25 °C) for 40–60 min. Afterward, the samples were rinsed with 50% ethanol. The root samples were photographed using a Leica stereomicroscope (Leica Microsystems, Heidelberg, Germany, Lecia 10447157).

### 4.5. Measurement of Physiological Indicators

To measure the salt tolerance ability between CRW3 and CRW13, the soluble sugar content, catalase (CAT) activity, and peroxidase (POD) activity were measured in root tip tissues using assay kits purchased from the Nanjing Jiancheng Bioengineering Institute. Soluble sugar content Kit (Nanjing Jiancheng Bioengineering Institute, Nanjing, China, Cat# A145-1-1) was determined via the anthrone colorimetric method. Fresh roots (0.1–0.2 g) were homogenized in distilled water (1:10, *w*/*v*), boiled for 10 min, and centrifuged at 4000 rpm. The supernatant was diluted 10-fold, and absorbance was read at 620 nm; results were expressed as mg g^−1^ FW. CAT activity Kit (Nanjing Jiancheng Bioengineering Institute, Nanjing, China, Cat# A007-1-1) was assayed using the ammonium molybdate colorimetric method. Samples were homogenized in pre-cooled PBS (0.1 M, pH 7.0–7.4, 1:9, *w*/*v*), centrifuged at 4000 rpm for 10 min, and the absorbance of the supernatant was measured at 405 nm; activity was expressed as U g^−1^ tissue. POD activity Kit (Nanjing Jiancheng Bioengineering Institute, Nanjing, China, Cat# A084-3-1) was determined by homogenizing root tips in PBS (0.1 M, pH 7.0–7.4, 1:9, *w*/*v*), centrifuging at 3500 rpm for 10 min, and measuring absorbance at 420 nm; activity was presented as U g^−1^ FW. All treatments included three biological replicates.

### 4.6. Measurement of Morphological Indicators

Three traits (shoot length, root length, and root number) were determined to evaluate the seedling growth of two common wild rice materials under salt stress. The dormancy-broken seeds were rinsed in 75% ethanol for 1–2 min, then soaked in a 2.5% NaClO solution with continuous shaking for 18–20 min, followed by 3–5 rinses with sterile distilled water and air-drying. Sterilized seeds were placed on 1/2 MS rooting medium (2.47 g half-strength MS powder and 4 g Phytagel per liter of double-distilled water, pH = 5.80) and germinated in darkness for 3–5 days. Germinated seedlings with consistent growth were transplanted into hydroponic boxes and cultivated to the two-leaf-one-heart stage.

The experiment included two treatment groups: a control group cultivated in deionized water and a salt-stress group treated with 100 mmol/L NaCl, both cultivated continuously for 7 days. Shoot length, root length, and root number were measured before treatment and on the 3rd and 7th days after treatment. Each treatment contained three biological replicates, and each replicate consisted of five homogeneous seedlings.

### 4.7. Semi-Thin Sectioning

Root samples from two wild rice species were collected on the 7th day under different salt stress concentrations and stored in FAA solution for 48 h. After fixation, samples were dehydrated through a series of ethanol concentrations, with each concentration maintained for at least 30 min. The dehydrated materials were then embedded using a Technovit 7100 embedding kit (Kulzer GmbHulzer, Hanau, Germany, Cat# 64709003) according to the manufacturer’s instructions. Embedded samples were sectioned on the Leica RM2235 microtome (Leica Microsystems, Heidelberg, Germany), stained with 1% toluidine blue O, and sealed with neutral balsam. The method was conducted in accordance with the previous study [36].

### 4.8. WE-CLSM Analysis

WE-CLSM analysis was used to examine the root tissues of two wild common rice varieties under salt-stress treatment. In this study, root samples subjected to different salt-stress concentrations were fixed in FAA solution for 48 h. Next, the samples were rehydrated using an ethanol gradient and stained with eosin B (C_20_H_6_N_2_O_9_Br_2_Na_2_, FW 624.1). The detailed protocol is described in our previous study [66]. The samples were scanned and examined using a Leica SP2 laser scanning confocal microscope (Leica Microsystems, Heidelberg, Germany) at a laser wavelength of 543 nm, and images were captured and recorded. Observations focused on morphological changes in root tip cell structures across the various treatment groups.

### 4.9. DNA Library Construction, Massive Resequencing, and Validation Analysis

Fresh leaf samples from two materials, CRW3 and CRW13, were collected and subjected to resequencing analysis. Sequencing libraries were prepared from genomic DNA of CRW3 and CRW13 and sequenced on an Illumina HiSeq™ 2500 platform (Illumina, Inc., San Diego, CA, USA). Biomarker Technologies (Beijing, China) conducted whole-genome resequencing with an average coverage of 45× per sample. The sequencing reads were aligned to the *Nipponbare* reference genome using the BWA software (version 0.7.17). Using the GATK software (version 4.1.3.0), polymorphic sites, including SNPs and InDels, were identified between the two materials. SnpEff (version 4.3p) was utilized to annotate SNPs and InDels between the two materials. SNP and InDel variants in the CDS region between CRW3 and CRW13 were further selected for genetic analysis. The CDS region was mainly composed of frameshift_variant, disruptive_inframe_deletion, and conservative_inframe_deletion. High and moderate effect types were selected for further analysis. Gene Ontology (GO) and KEGG pathway enrichment analyses were conducted using OmicShare Tools v3.0 (Gene Denovo Biotechnology Co., Ltd., Guangzhou, China).

### 4.10. RT-qPCR Analysis

RT-qPCR analysis was conducted to examine how roots respond to salt stress. A total of nine candidate genes were selected in this study (Table S7). The AG First Strand cDNA Synthesis Kit was used to perform the reverse transcription reaction. RT-qPCR was performed under the following conditions: 30 s at 95 °C, followed by 40 cycles, each consisting of 5 s at 95 °C for denaturation and 20 s at 58 °C for annealing and extension. The RT-qPCR experiment was conducted using the LightCycler 480 system (Roche Diagnostics GmbH, Mannheim, Germany) and SYBR Green Permix Pro Taq HS qPCR Tracking Kit (Accurate Biology, Changsha, China, Cat# AG11733-S). All qRT-PCR reactions were conducted in triplicate, with results calculated via the 2^−ΔΔCt^ method [67]. The rice actin gene served as an internal control to normalize expression levels.

### 4.11. Data Analysis

Experimental data were analyzed using Microsoft Excel 2019. Statistical significance was assessed using IBM SPSS Statistics 27, employing one-way ANOVA and Duncan’s test (*p* < 0.05). A graphical representation of the results was created using GraphPad Prism 9.0.

## 5. Conclusions

In this study, we evaluated the salt-stress tolerance of 13 common wild rice accessions collected from Gaozhou City, Guangdong Province. Among these, two accessions, CRW3 and CRW13, were identified as the most salt-tolerant and salt-sensitive, respectively. Analyses of the seeding and tiller stems demonstrated that CRW3 exhibited significantly higher salt tolerance than CRW13. Cytological observations further revealed that NaCl treatment had a negligible effect on the root tip and cortical cells of CRW3. Moreover, comparative resequencing analysis identified 2390 differentially expressed genes between CRW3 and CRW13. The salt-stress-related genes were subsequently validated by RT-qPCR analysis. These findings indicate that CRW3 showed a strong salt-tolerant ability and may be an important salt-tolerant material.

## Figures and Tables

**Figure 1:**
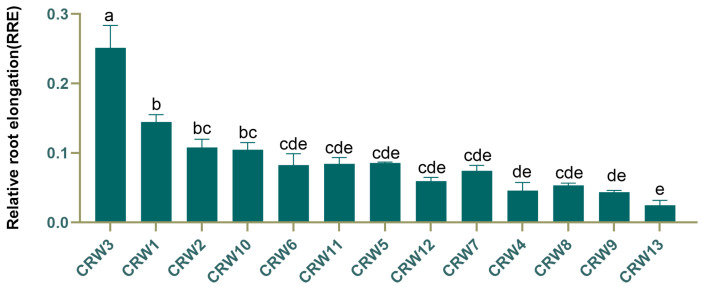
Comparison of RRE in various common wild rice varieties in Gaozhou City, Guangdong Province. All materials were treated with 150 mmol/L NaCl for 7 days. Values are presented as mean ± SE (n = 3 biological replicates). Each treatment consisted of three biological replicates. For each replicate, five tillers were sampled, and five roots from each tiller were measured. Statistical significance was assessed using one-way ANOVA followed by Duncan’s multiple range test. Different lowercase letters indicate significant differences among different varieties (*p* < 0.05).

**Figure 2:**
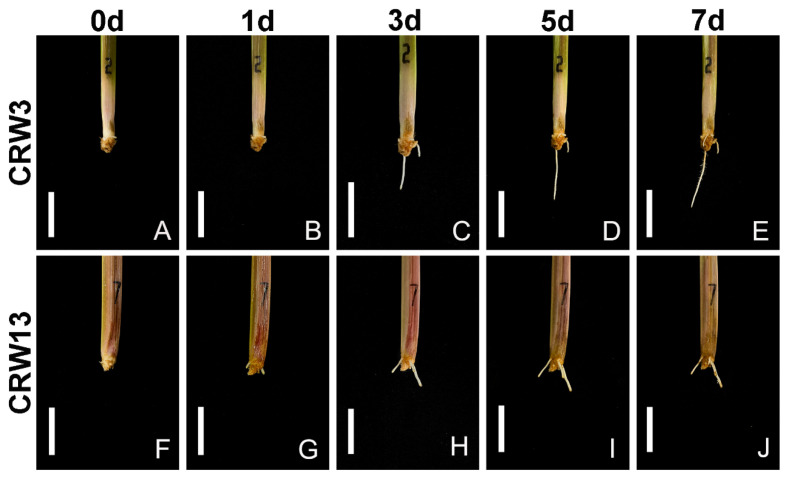
Comparison of root morphologies between CRW3 and CRW13 at 150 mmol/L NaCl. (**A**–**E**) The tiller stem of CRW3 under NaCl stress at various days. (**F**–**J**) The tiller stem of CRW13 under NaCl stress on different days. Bars = 2 cm.

**Figure 3:**
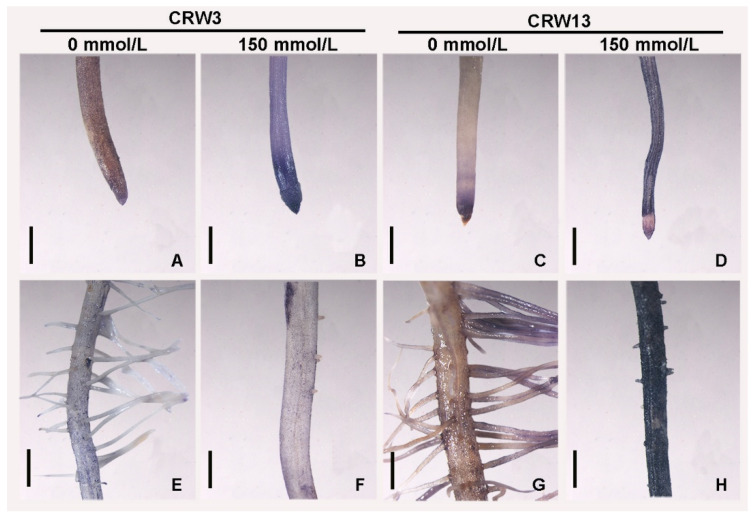
Comparison of NBT staining in root tips and lateral root regions of CRW3 and CRW13 under both 0 and 150 mmol/L NaCl stress. (**A**) NBT staining of the root tip in CRW3 without NaCl on the 7th day. (**B**) NBT staining of the root tip in CRW3 with 150 mmol/L NaCl on the 7th day. (**C**) NBT staining of the root tip in CRW13 without NaCl after 7 days. (**D**) NBT staining of the root tip in CRW13 with 150 mmol/L NaCl on the 7th day. (**E**) NBT staining of the lateral root region in CRW3 without NaCl on the 7th day. (**F**) NBT staining of the lateral root region in CRW3 with 150 mmol/L NaCl on the 7th day. (**G**) NBT staining of the lateral root region in CRW13 without NaCl on the 7th day. (**H**) NBT staining of the lateral root region in CRW13 with 150 mmol/L NaCl on the 7th day. Bars = 1 mm.

**Figure 4:**
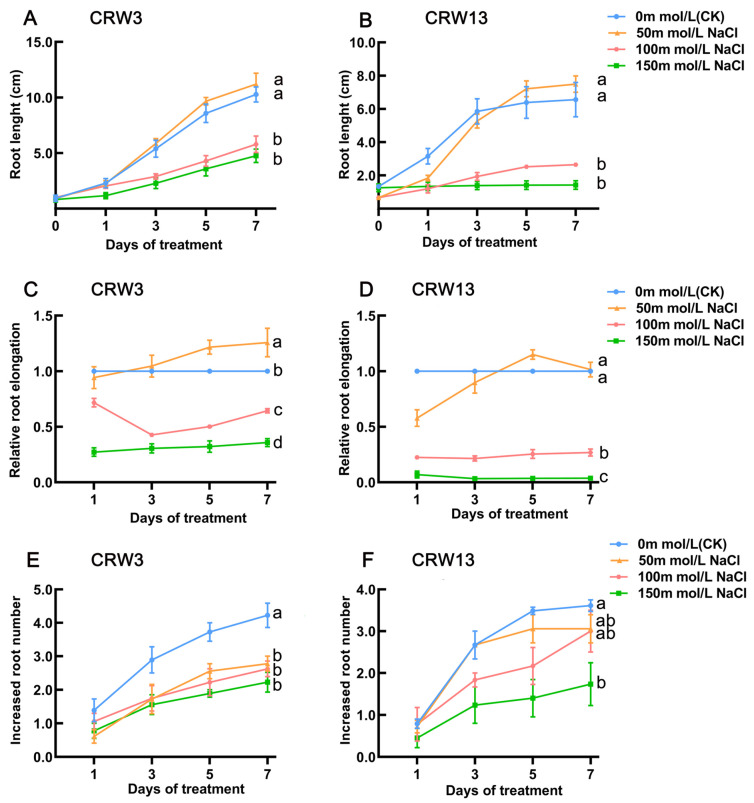
Comparison of root length, relative root elongation, and increased root number between CRW3 and CRW13 under different NaCl concentrations. (**A**) Root length of CRW3 across different NaCl concentrations from day 0 to the 7th day. (**B**) Root length of CRW13 across different NaCl concentrations from day 0 to the 7th day. (**C**) Relative root elongation of CRW3 across different NaCl concentrations from day 0 to the 7th day. (**D**) Relative root elongation of CRW13 across different NaCl concentrations from day 0 to the 7th day. (**E**) Increased root number of CRW3 after 7 days of treatment at different NaCl concentrations. (**F**) Increased root number of CRW13 after 7 days of treatment at different NaCl concentrations. Values are presented as mean ± SE (n = 3 biological replicates). Each treatment consisted of three biological replicates. For each replicate, five tillers were sampled, and five roots from each tiller were measured. Statistical significance was assessed using one-way ANOVA followed by Duncan’s multiple range test. Different lowercase letters indicate significant differences among NaCl treatments (*p* < 0.05).

**Figure 5:**
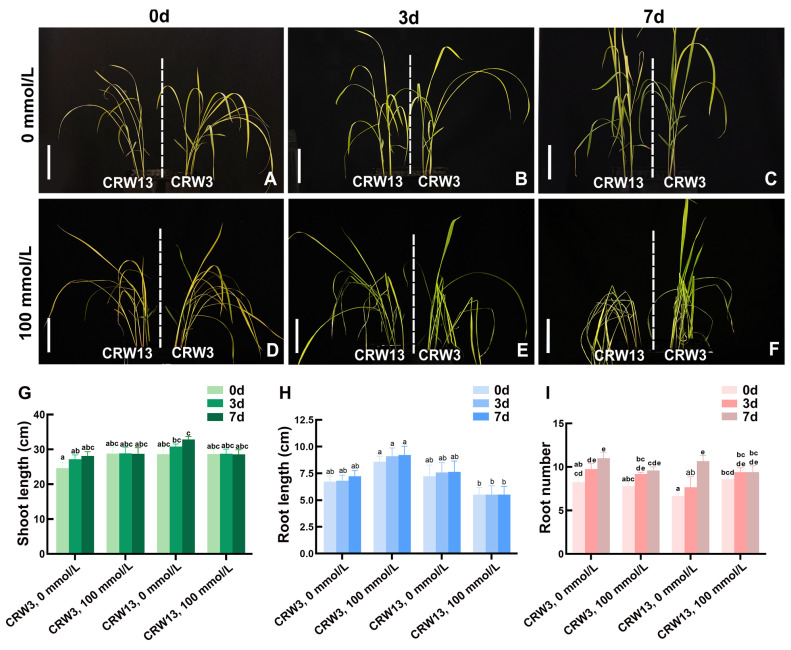
Comparison of seedings of CRW13 and CRW3 under salt-stress treatment. (**A**) Phenotypic comparison of CRW3 and CRW13 at the seedling stage without NaCl stress. Scale bar = 10 cm. (**B**) Phenotypic comparison of seedlings without NaCl stress on the 3rd day. Scale bar = 10 cm. (**C**) Phenotypic comparison of seedlings without NaCl stress on the 7th day. Scale bar = 10 cm. (**D**) Phenotypic comparison of seedlings under 100 mmol/L NaCl stress. Scale bar = 10 cm. (**E**) Phenotypic comparison of seedlings under 100 mmol/L NaCl stress on the 3rd day. Scale bar = 10 cm. (**F**) Phenotypic comparison of seedlings under 100 mmol/L NaCl stress on the 7th day. Scale bar = 10 cm. (**G**) Comparison of shoot length of CRW3 and CRW13 under 0 and 100 mmol/L NaCl stress at 0, 3, and 7 days. (**H**) Root length comparison under the same conditions and times. (**I**) Comparison of root numbers for CRW3 and CRW13 under 0 and 100 mmol/L NaCl stress at 0, 3, and 7 days. Values are presented as mean ± SE (n ≥ 3 biological replicates). Each treatment consisted of three biological replicates. For each replicate, five tillers were sampled, and five roots from each tiller were measured. Statistical significance was assessed using one-way ANOVA followed by Duncan’s multiple range test. Different lowercase letters indicate significant differences among NaCl treatments and two materials (*p* < 0.05).

**Figure 6:**
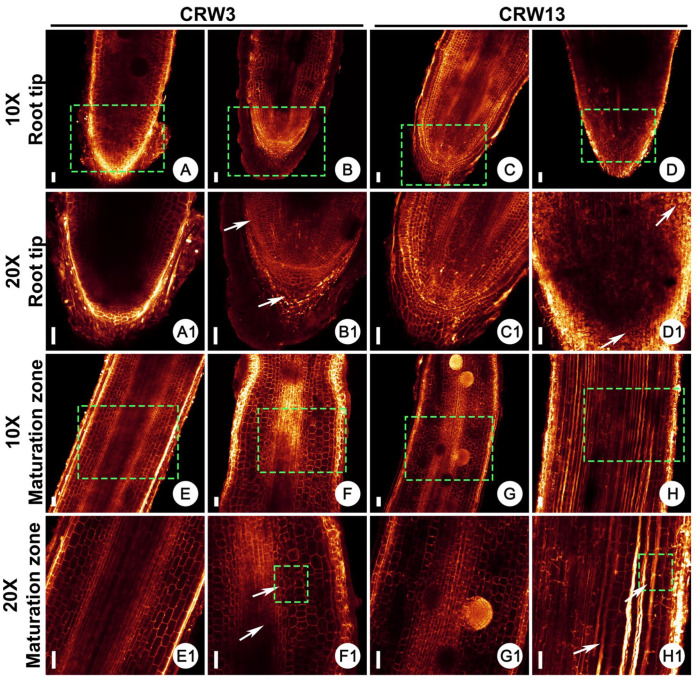
Comparison of the root tip cell morphology between CRW3 and CRW13 under 0 and 150 mmol/L NaCl treatments. (**A**–**D**) Comparison of the root tip cells of CRW3 and CRW13 under 0 and 150 mmol/L NaCl. (**A**) Root tip cells of CRW3 on the 7th day with no NaCl treatment. (**B**) Root tip cells of CRW3 on the 7th day under 150 mmol/L NaCl. (**C**) Root tip cells of CRW13 on the 7th day with no NaCl treatment. (**D**) Root tip cells of CRW13 on the 7th day under 150 mmol/L NaCl. (**E**–**H**) Comparison of the maturation zone cells of CRW3 and CRW13 under 0 and 150 mmol/L NaCl. (**E**) Maturation zone cells of CRW3 on the 7th day with no NaCl treatment. (**F**) Maturation zone cells of CRW3 on the 7th day under 150 mmol/L NaCl. (**G**) Maturation zone cells of CRW13 on the 7th day under no NaCl treatment. (**H**) Maturation zone cells of CRW13 on the 7th day under 150 mmol/L NaCl. (**A1**–**D1**) Detailed diagram of the root tip cells indicated by green dashed boxes in (**A**–**D**) under 20× magnification. (**E1**–**H1**) Detailed diagram of the maturation zone cell areas indicated by green dashed boxes in (**E**–**H**) at 20× magnification. White arrows indicate root tip cell abnormalities in CRW3 and CRW13 under 150 mmol/L NaCl treatment compared with no NaCl treatment. Scale bars = 50 μm.

**Figure 7:**
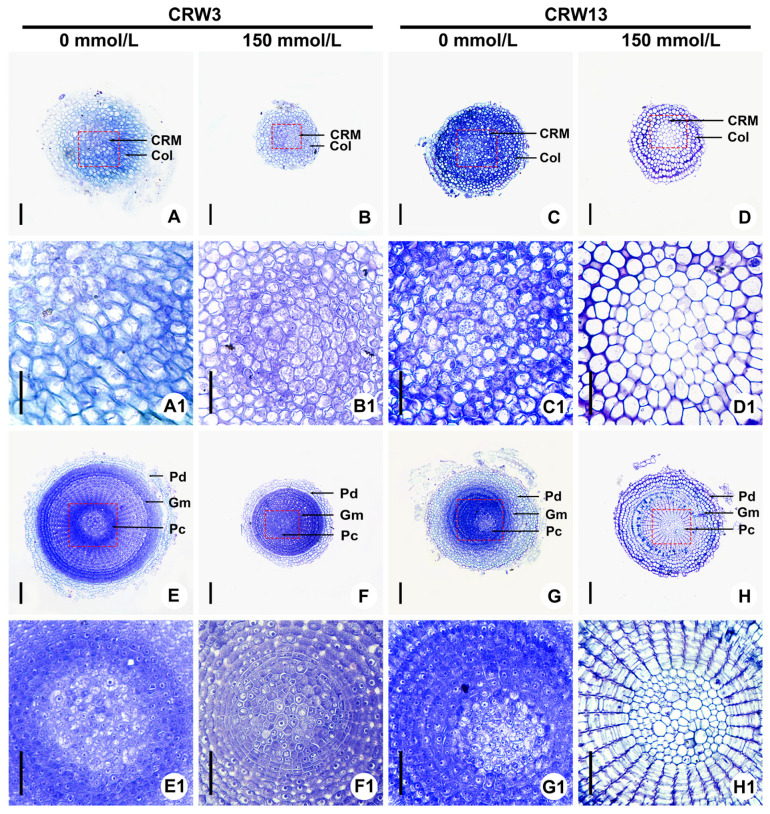
Comparison of root cap and meristematic zone cells between CRW3 and CRW13 on the 7th day under 0 and 150 mmol/L NaCl treatment. (**A**–**D**): Comparison of root cap cells between CRW3 and CRW13 under 0 and 150 mmol/L NaCl treatment, respectively. (**A**) Cross-section of root cap cells of CRW3 under 0 mmol/L NaCl on the 7th day. (**B**) Cross-section of root cap cells of CRW3 under 150 mmol/L NaCl on the 7th day. (**C**) Cross-section of root cap cells of CRW13 under 0 mmol/L NaCl on the 7th day. (**D**) Cross-section of root cap cells of CRW13 under 150 mmol/L NaCl on the 7th day. (**E**–**H**) Comparison of root meristematic zone cells between CRW3 and CRW13 under 0 and 150 mmol/L NaCl treatment, respectively. (**E**) Cross-section of root meristematic zone cells of CRW3 under 0 mmol/L NaCl on the 7th day. (**F**) Cross-section of root meristematic zone cells of CRW3 under 150 mmol/L NaCl on the 7th day. (**G**) Cross-section of root meristematic zone cells of CRW13 under 0 mmol/L NaCl on the 7th day. (**H**) Cross-section of root meristematic zone cells of CRW13 under 150 mmol/L NaCl on the 7th day. (**A1**–**D1**) Magnified views of the red dashed box areas in (**A**–**D**). (**E1**–**H1**) Magnified views of the root meristematic zone corresponding to the red dashed box areas in (**E**–**H**). Red dashed boxes indicate the magnified details of (**A**–**H**). Scale bars = 60 μm in (**A**–**H**), 40 μm in (**A1**–**H1**). CRM: Central Root Meristem; Col: Columella; Pd: Protoderm; Gm: Ground Meristem; Pc: Procambium.

**Figure 8:**
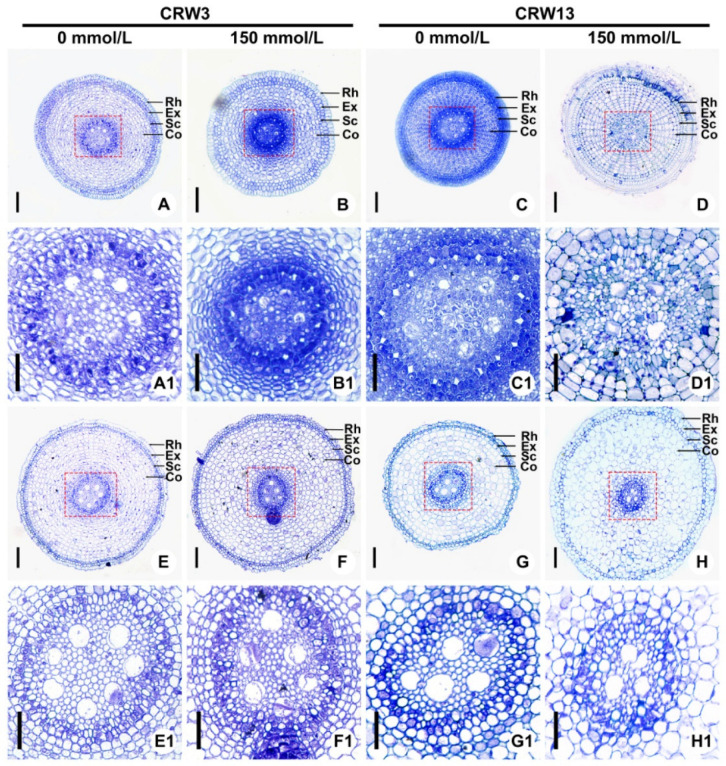
Comparison of elongation-zone and mature-zone cells between CRW3 and CRW13 on the 7th day under 0 and 150 mmol/L NaCl treatments. (**A**–**D**) Comparison of elongation-zone cells between CRW3 and CRW13 under 0 and 150 mmol/L NaCl treatment, respectively. (**A**) Cross-section of elongation-zone cells of CRW3 at 0 mmol/L NaCl on the 7th day. (**B**) Cross-section of elongation-zone cells of CRW3 at 150 mmol/L NaCl on the 7th day. (**C**) Cross-section of elongation-zone cells of CRW13 at 0 mmol/L NaCl on the 7th day. (**D**) Cross-section of elongation-zone cells of CRW13 at 150 mmol/L NaCl on the 7th day. (**E**–**H**): Comparison of mature-zone cells of CRW3 and CRW13 under 0 and 150 mmol/L NaCl treatment, respectively. (**E**) Cross-section of mature-zone cells of CRW3 at 0 mmol/L NaCl on the 7th day. (**F**) Cross-section of mature-zone cells of CRW3 under 150 mmol/L NaCl on the 7th day. (**G**) Cross-section of mature-zone cells of CRW13 under 0 mmol/L NaCl on the 7th day. (**H**) Cross-section of mature-zone cells of CRW13 under 150 mmol/L NaCl on the 7th day. (**A1**–**D1**) Stelar cell structure in the root elongation zone, corresponding to the red dashed box areas in (**A**–**D**). (**E1**–**H1**) Stelar cell structure in the root mature zone, corresponding to the red dashed box areas in (**E**–**H**). Red dashed boxes indicate the magnified details of (**A**–**H**). Scale bars = 60 μm in (**A**–**H**), 40 μm in (**A1**–**H1**). Rh: rhizodermis; Ex: exodermis; Sc: sclerenchyma; Co: cortex.

**Figure 9:**
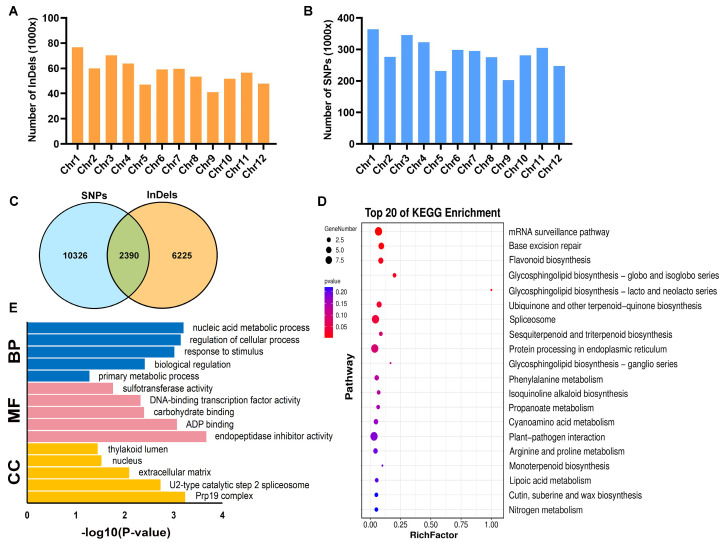
Genomic variation of the CRW3 and CRW13. (**A**) Number of InDels variations between CRW3 and CRW13 on each chromosome. (**B**) Number of SNP variations between CRW3 and CRW13 on each chromosome. (**C**) Venn diagram analysis of differential SNPs and InDels between CRW3 and CRW13. (**D**) KEGG pathways are significantly enriched for differentially expressed genes between CRW3 and CRW13. (**E**) GO enrichment analysis of genomic variation genes of CRW3 and CRW13.

**Figure 10:**
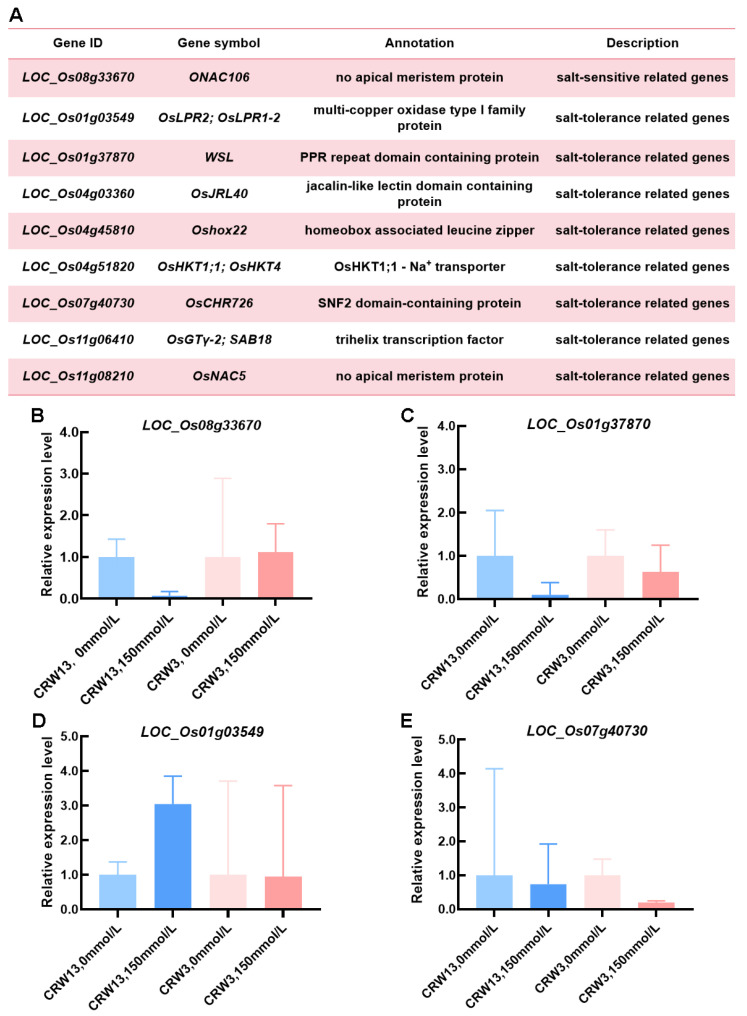
RT-qPCR confirmation of salt-related genes in CRW3 and CRW13 between 0 mmol/L and 150 mmol/L NaCl. (**A**) Annotation of nine salt-related genes. (**B**) RT-qPCR confirmation of *LOC_Os08g33670* in CRW3 and CRW13 between 0 mmol/L and 150 mmol/L NaCl. (**C**) RT-qPCR confirmation of *LOC_Os01g37870* in CRW3 and CRW13 between 0 mmol/L and 150 mmol/L NaCl. (**D**) RT-qPCR confirmation of *LOC_Os01g03549* in CRW3 and CRW13 between 0 mmol/L and 150 mmol/L NaCl. (**E**) RT-qPCR confirmation of *LOC_Os07g40730* in CRW3 and CRW13 between 0 mmol/L and 150 mmol/L NaCl. Each treatment contained three biological replicates, and each biological replicate was analyzed with three technical replicates.

## Data Availability

The data are contained within the article and its Supplementary Materials.

## Supplementary Materials

The following supporting information can be downloaded at: https://www.mdpi.com/article/10.3390/plants15152247/s1, Table S1: Comparison of the soluble sugar content, CAT activity, and POD activity between CRW3 and CRW13; Table S2: Comparison of the root tip anatomical parameters of common wild rice under salt stress; Table S3: GO enrichment analysis of biological process category between CRW3 and CRW13; Table S4: GO enrichment analysis of molecular function category between CRW3 and CRW13; Table S5: GO enrichment analysis of cellular component category between CRW3 and CRW13; Table S6: List of the salt-stress-related genes; Table S7: List of primers used for RT-qPCR analysis; Figure S1: RT-qPCR confirmation of eight salt-related genes in CRW3 and CRW13 between 0 mmol/L and 150 mmol/L NaCl.
