# Supplementary material for: Cytological and Gene Expression Analysis Reveal Salt Resistance in Common Wild Rice (Oryza rufipogon Griff.): A Case for Root Tissue Resistance
Source: Plants (Basel). 2026 Jul 23;15(15):2247. doi: 10.3390/plants15152247 (PMC13468594; doi:10.3390/plants15152247)
Supplement: Supplementary file 1 [file plants-15-02247-s001.zip › Figure S1.pdf]

A

Gene ID	Gene symbol	Annotation	Description
LOC_Os03g13140	*OsPgb1.1*	Non-symbiotic hemoglobin 2	Salt-tolerance related genes
LOC_Os05g48700	*OsGA2ox8*	Gibberellin 2-beta-dioxygenase	Salt-tolerance related genes
LOC_Os06g27760	*OsMSRB1.1*	Peptide methionine sulfoxide reductase msrB	Salt-tolerance related genes
LOC_Os07g05940	*OsNCED4*	9-cis-epoxycarotenoid dioxygenase 1	Salt-tolerance related genes
LOC_Os02g52780	*OsbZIP23*	bZIP transcription factor	Salt-sensitive related genes
LOC_Os07g48130	*OsHAK9*	Potassium transporter	Salt-sensitive related genes
LOC_Os09g13570	*OsbZIP71*	Expressed protein	Salt-sensitive related genes
LOC_Os12g42280	*OsNCED5*	9-cis-epoxycarotenoid dioxygenase	Salt-sensitive related genes

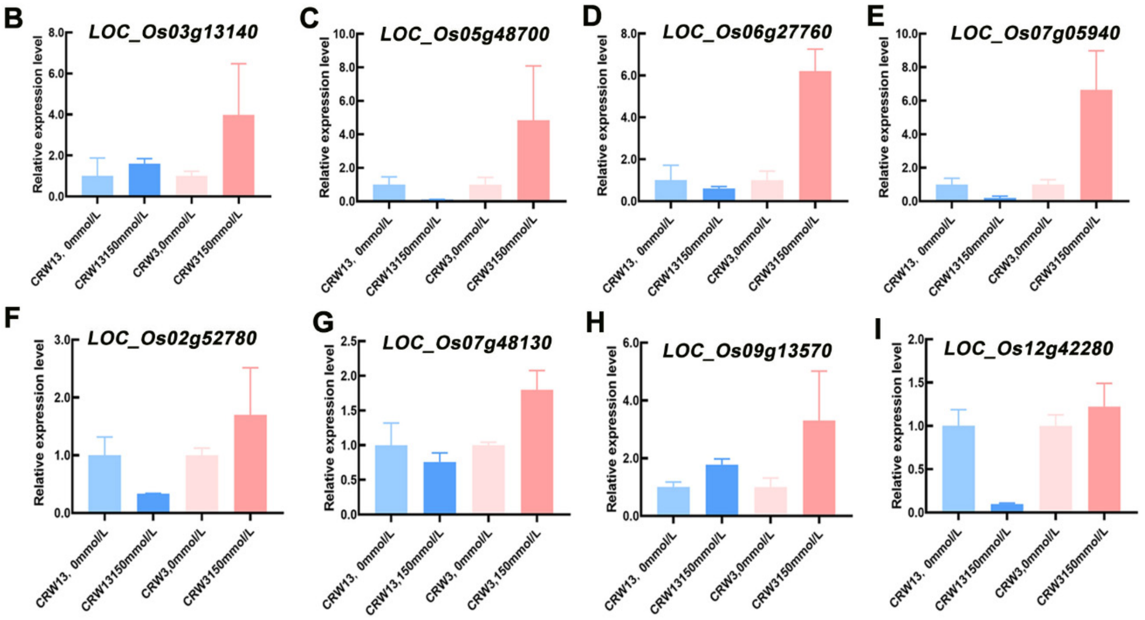

Figure S1. The RT-qPCR confirmation of salt-related functionalized genes in CRW3 and CRW13 between 0 mmol/L and 150 mmol/L NaCl. (**A**) Annotation of eight salt-related genes. (**B**) The RT-qPCR confirmation of *LOC_Os03g13140* in CRW3 and CRW13 between the 0 mmol/L and 150 mmol/L NaCl. (**C**) The RT-qPCR confirmation of *LOC_Os05g48700* in CRW3 and CRW13 between the 0 mmol/L and 150 mmol/L NaCl. (**D**) The RT-qPCR confirmation of *LOC_Os06g27760* in CRW3 and CRW13 between the 0 mmol/L and 150 mmol/L NaCl. (**E**) The RT-qPCR confirmation of *LOC_Os07g05940* in CRW3 and CRW13 between the 0 mmol/L and 150 mmol/L NaCl. (**F**) The RT-qPCR confirmation of *LOC_Os02g52780* in CRW3 and CRW13 between the 0 mmol/L and 150 mmol/L NaCl. (**G**) The RT-qPCR confirmation of *LOC_Os07g48130* in CRW3 and CRW13 between the 0 mmol/L and 150 mmol/L NaCl. (**H**) The RT-qPCR confirmation of *LOC_Os09g13570* in CRW3 and CRW13 between the 0 mmol/L and 150 mmol/L NaCl. (**I**) The RT-qPCR confirmation of *LOC_Os12g42280* in CRW3 and CRW13 between the 0 mmol/L and 150 mmol/L NaCl. Each treatment contained three biological replicates, and each biological replicate was analyzed with three technical replicates.
